# Association between dyslipidemia and serum uric acid levels in Korean adults: Korea National Health and Nutrition Examination Survey 2016-2017

**DOI:** 10.1371/journal.pone.0228684

**Published:** 2020-02-14

**Authors:** Minkook Son, Jeongkuk Seo, Sung Yang

**Affiliations:** 1 Department of Biomedical Science and Engineering, Gwangju Institute of Science and Technology, Gwangju, Republic of Korea; 2 Department of Internal Medicine, Armed Forces Goyang Hospital, Goyang-si, Republic of Korea; 3 School of Mechanical Engineering, Gwangju Institute of Science and Technology, Gwangju, Republic of Korea; Università degli Studi di Milano, ITALY

## Abstract

**Objectives:**

Despite the growing pieces of evidence linking hyperuricemia with metabolic syndrome and cardiovascular disease, the relationship between dyslipidemia and serum uric acid has not yet been established. This study aimed to investigate the association between individual components of dyslipidemia and serum uric acid by using the nationally representative Korea National Health and Nutrition Examination Survey 2016–2017.

**Methods:**

A total of 8,722 participants (age ≥ 19 years) without missing values were analyzed for this study. Serum uric acid levels according to the presence of individual dyslipidemia components were calculated using multivariable-adjusted general linear models (GLM). Odds ratios of individual dyslipidemia components to hyperuricemia were calculated using unadjusted and multivariable-adjusted logistic regression analysis.

**Results:**

A total of 1,061 participants were identified as having hyperuricemia, with a prevalence of 12.2%. Multivariable-adjusted GLM demonstrated a significant trend between individual dyslipidemia components and serum uric acid levels (P < 0.05). A positive association between the numbers of dyslipidemia components and the increments of serum uric acid levels was also observed (P < 0.001). In multivariable-adjusted logistic regression analysis, odds ratios (OR) and 95% confidence interval (CI) of all dyslipidemia components to hyperuricemia were shown to be statistically significant (P < 0.05). When further adjusted for the combined components themselves, each 10 mg/dL increments of total cholesterol (OR 1.053; 95% CI 1.028–1.079), triglycerides (OR 1.017; 95% CI 1.009–1.026) and HDL-C (OR 0.804; 95% CI 0.729–0.887), retained significant correlation with hyperuricemia.

**Conclusion:**

Our study demonstrated that the dyslipidemia components of serum total cholesterol, triglycerides and LDL-C levels are positively associated with serum uric acid levels, whereas serum HDL-C levels are inversely related. Further complementary studies regarding other lipid parameters are needed to confirm the accurate association between dyslipidemia and serum uric acid levels.

## Introduction

Serum uric acid levels are known to be strongly linked with stroke [1], coronary artery disease [2] as well as hypertension [3] and metabolic syndrome [4]. However, the definite role of serum uric acid in these diseases is not yet established, due to its association with numerous other risk factors such as diet, obesity and dyslipidemia [5]. Emerging shreds of evidence show a rapidly increasing the prevalence of hyperuricemia in the international communities [6, 7], calling for investigations on the impact of serum uric acid in the pathogenesis and the prevalence of cardiovascular disease and metabolic syndrome.

Although the risk factors for metabolic syndrome and cardiovascular disease are being thoroughly studied, only a few studies to date have linked dyslipidemia to serum uric acid, including those investigated with the adult population of Bangladeshi [8], India [9], Italy [10], China [11], and USA [5]. However, despite the high prevalence (11.4%) of hyperuricemia in Korean population [12], information is lacking on the association of dyslipidemia with serum uric acid within the community. Considering the results of the aforementioned studies being insufficiently adjusted for the various laboratory & clinical confounders, in this study, we aimed to investigate the independent relationship between individual components of dyslipidemia and serum uric acid, with extensive adjustments for possible confounders, using the representative datasets of the Korean population.

## Materials and methods

### Study population

**Fig 1:**
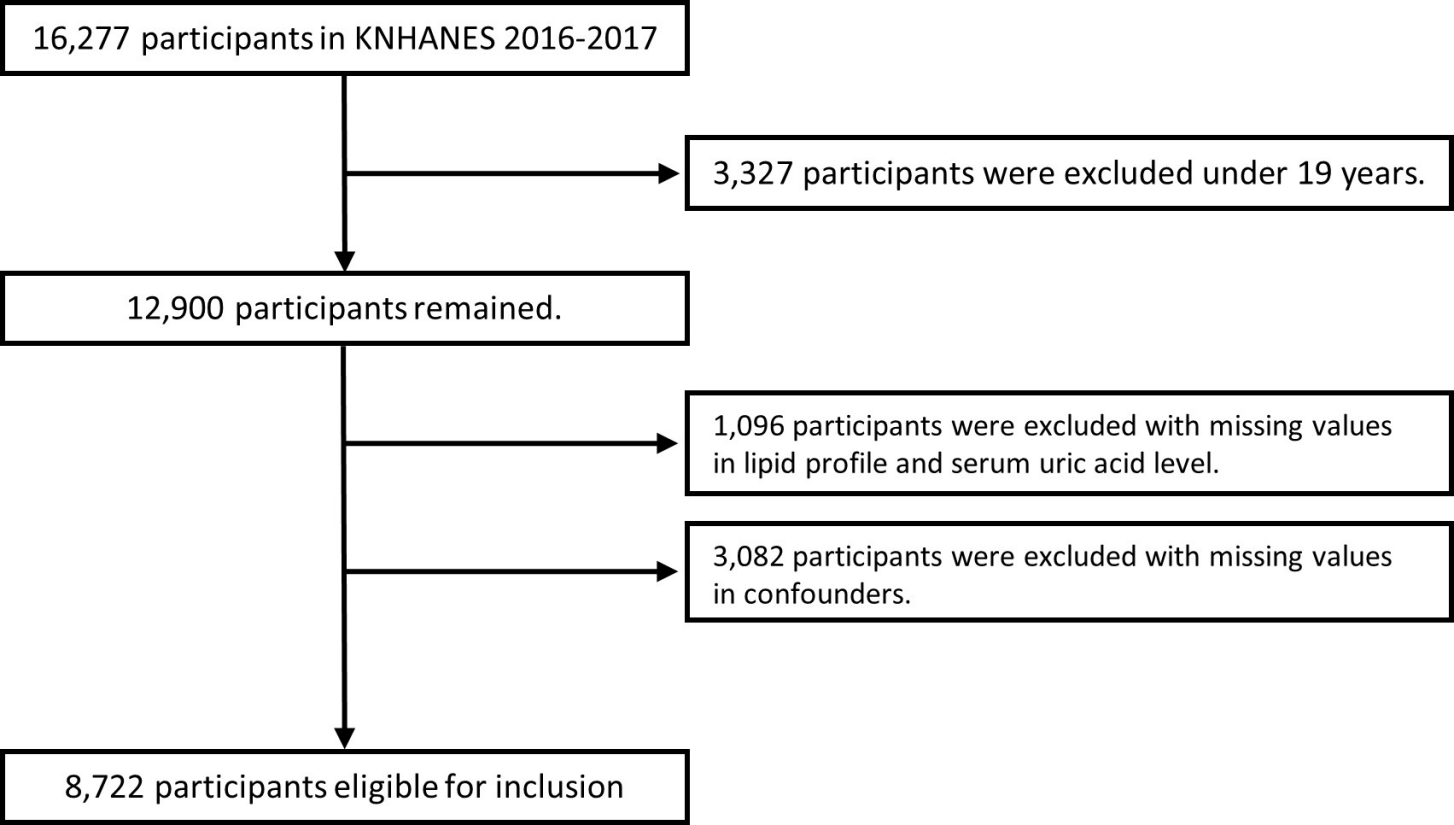
Flow diagram of the present study. Confounders include age, sex, waist circumference, BMI, hemoglobin, BUN, GFR, HTN, DM, smoking, alcohol consumption, regular exercise and dyslipidemia medication.

This study analyzed data from the seventh Korea National Health And Nutrition Examination Survey (KNHANES Ⅶ; 2016–2017) [13]. The KNHANES is a cross-sectional survey and a nationally representative database for Korean population, controlled by the Korea Centers for Disease Control and Prevention (KCDC). The survey uses a stratified multi-stage probability sampling method. KNHANES consists of health interviews, health examinations and nutritional surveys. Further information regarding study design and data variables can be found on the relevant website [14]. From the total number of 16,277 participants in KNHANES Ⅶ, 8,722 (male 3,704 [42.4%]) participants over the age of 19 without missing values were analyzed for this study. More information about participants and study design can be found in Fig 1.

Informed consent was obtained from all participants for voluntary participation and the KNHANES was approved by the Institutional Review Board of KCDC. The protocol of this study was approved by the Institutional Review Board of Gwangju Institute of Science and Technology (20190705-EX-01-02), which waived the requirement for informed consent in regard to the anonymized data analyzed retrospectively. This study was performed based on the Declaration of Helsinki.

### Demographic characteristics and survey on the status of lifestyle

Demographic data and medical history were obtained from self-reported questionnaires and personal interviews with trained staff. Level of education was divided into elementary school graduates or lower, middle school graduates, high school graduates, and college graduates or higher. Status regarding smoking was divided into 3 groups: (a) having smoked more than 5 packs of cigarettes and current smoking was defined as ‘current smokers’, (b) having smoked more than 5 packs of cigarettes and current non-smoking was defined as ‘ex-smokers’, and (c) those with less than 5 packs of cigarette smoking as ‘never smokers’. Alcohol consumption status was divided into 2 groups which are as follows: (a) ‘non-alcohol consumer’ with no alcohol consumption for the past year or consumption less than once a month, and (b) ‘alcohol consumer’ with alcohol consumption more than once a month. In regard to physical exercise, subjects were categorized into 2 groups: (a) ‘regular exercise’ group engaging in ≥ 150 minutes of moderately hard exercise per week or in ≥ 75 minutes of hard exercise per week or mixed exercise equivalent to the above level (1 minute of hard exercise equivalent to 2 minutes of moderately hard exercise) and (b) ‘non-regular exercise’ group with activity less than aforementioned level.

### Anthropometric and laboratory measurements

Height and body weight were measured by a standardized protocol and body mass index (BMI) were calculated using weight (kg) per square of height (m^2^). Waist circumference was defined as the circumference measured at the midline of lower rib margin to iliac crest. Blood pressure was measured 3 times by trained nurses using a mercury sphygmomanometer (Baumanometer, WA Baum Co., NY, USA) in a seated position with arm supported at heart level after 5 minutes of rest.

All participants fasted for at least 8 hours before blood sampling. The samples were processed immediately and then refrigerated for transportation to the NeoDin Medical Institute (Seoul, Korea). All laboratory measurements were performed by the NeoDin Medical Institute. Further information for laboratory measurements can be found on the relevant website [14]. The Friedewald formula was used for the calculation of low density lipoprotein cholesterol (LDL-C) [15]. If triglyceride was over 400 mg/dL, values of direct measurement for LDL cholesterol levels were used. Non high density lipoprotein cholesterol (Non-HDL-C) is calculated as total cholesterol minus HDL-C [16]. Estimated glomerular filtration rate (eGFR) were calculated using the modification of diet in renal disease (MDRD) equation [17].

### Definition of clinical variables

Hypertensive status was categorized into 3 groups: (a) 'hypertension' for systolic blood pressure (SBP) ≥140 mmHg or diastolic blood pressure (DBP) ≥ 90 mmHg or on anti-hypertensive medication, (b) 'pre-hypertensive' for 120 mmHg ≤ SBP < 140mmHg or 80 mmHg ≤ DBP < 90mmHg, (c) and 'normal' for SBP < 120mmHg and DBP < 80mmHg. Diabetic status was categorized into 3 groups: (a) 'diabetes' for fasting blood sugar (FBS) ≥ 126 mg/dL or diagnosed or on medication for diabetes, (b) 'pre-diabetes' for 100 mg/dL ≤ FBS < 126mg/dL, (c) and 'normal' for FBS < 100mg/dL. Status regarding hypercholesterolemia, hypertriglyceridemia, low HDL-C and high LDL-C were categorized into 2 groups using criteria for the diagnosis of dyslipidemia in Koreans [18] and the cut-off values were designated as ≥ 240 mg/dL, ≥ 200 mg/dL, ≤ 40 mg/dL and ≥ 160 mg/dL respectively. Additionally, high level of non-HDL-C was set as ≥ 160 mg/dL [16]. Hyperuricemia was defined as serum uric acid level ≥ 7.0 mg/dL in men and ≥ 6.0 mg/dL in women [19].

### Statistical analysis

Statistical procedures were conducted to reflect the complex sampling design and sampling weights of KNHANES Ⅶ. Data for continuous variables are presented as weighted means ± standard errors (SE) and data for categorical variables are presented as the number of cases with a weighted percentage. Characteristics of the participants with hyperuricemia were compared with those of participants without hyperuricemia using two independent sample Student’s t-tests for continuous variables and Chi-square test for categorical variables. The serum uric acid levels according to the presence of dyslipidemia were calculated using general linear models (GLM) with adjustments for multiple confounders and were compared by Bonferroni methods. Additionally, considering the differing serum uric acid levels between both gender, serum uric acid levels were calculated using GLM with the stratification of male and female groups. Unadjusted and multivariable-adjusted logistic regression analysis between each dyslipidemia components and hyperuricemia were performed. For multivariable-adjusted analysis, 4 models with progressive degrees of adjustment were utilized. Model 1 was adjusted for age and sex. Model 2 was further adjusted for age, sex and BMI. Model 3 was adjusted for age, sex, BMI, waist circumference, hemoglobin, blood urea nitrogen (BUN), glomerular filtration rate (GFR), hypertension, diabetes, smoking status, intake of alcohol and regular exercise, with model 4 further adjusted for the administration of dyslipidemia medication. All statistical analyses were performed with the complex sample procedures of SPSS software version 20 (IBM, SPSS Inc., NY, USA), and graphs in this study were drawn by Excel 2016 (Microsoft, USA). The p-value < 0.05 was considered to be statistically significant.

## Results

### Demographic characteristics of study participants

The baseline characteristics of study participants according to the presence of hyperuricemia are summarized in Table 1. Among a total of 8,722 participants, 1,061 participants were identified as the hyperuricemia group (prevalence 12.2%, uric acid level designated as ≥ 7 mg/dL in men, ≥ 6 mg/dL in women) whereas 7,661 participants were assigned to non-hyperuricemia group. Compared with non-hyperuricemia group, sexual proportion (male to female); BMI; waist circumference; SBP and DBP; hemoglobin; BUN; GFR; high sensitivity C-reactive protein; and serum uric acid levels tended to be elevated in the hyperuricemia group whereas average age tended to be lower. Total cholesterol, triglycerides, LDL-C and non-HDL-C levels were elevated with hyperuricemia whereas serum HDL-C levels were decreased. Participants with increased cumulative numbers of dyslipidemia components were significantly prevalent in the hyperuricemia group (P < 0.001). History of hypertension; diabetes; current smoking status; intake of alcohol; and regular exercise were also reported to be more frequent in the hyperuricemia group (P < 0.05).

**Table 1:** Characteristics of participants by the presence of hyperuricemia.

Total (n = 8,722)	Without hyperuricemia (n = 7,661)	With hyperuricemia (n = 1,061)	p-value^a^
**Age (years)**	47.72 ± 0.32	45.39 ± 0.64	< 0.001
**Sex (male)**	2,986 (43.9)	718 (74.9)	< 0.001
**Education (n)**			0.064
elementary school or lower	1,703 (15.9)	219 (12.9)	
middle school	771 (9.0)	108 (8.3)	
high school	2,320 (33.3)	339 (35.8)	
college or higher	2,867 (41.8)	395 (43.0)	
**BMI (kg/m**^**2**^**)**	23.62 ± 0.06	25.89 ± 0.14	< 0.001
**Waist circumference (cm)**	81.25 ± 0.18	88.39 ± 0.37	< 0.001
**SBP (mmHg)**	116.90 ± 0.26	121.59 ± 0.59	< 0.001
**DBP (mmHg)**	75.18 ± 0.16	78.93 ± 0.44	< 0.001
**Hemoglobin (g/dL)**	14.07 ± 0.02	14.92 ± 0.06	< 0.001
**BUN (mg/dL)**	13.77 ± 0.06	15.21 ± 0.19	< 0.001
**GFR (mL/min/1.73m**^**2**^**)**	97.17 ± 0.32	87.44 ± 0.69	< 0.001
**Fasting glucose (mg/dL)**	98.95 ± 0.33	100.29 ± 0.67	0.053
**hs-CRP (mg/L)**	1.15 ± 0.03	1.62 ± 0.08	< 0.001
**Serum uric acid (mg/dL)**	4.75 ± 0.01	7.52 ± 0.03	< 0.001
**Total cholesterol (mg/dL)**	192.63 ± 0.51	201.95 ± 1.61	< 0.001
**Triglycerides (mg/dL)**	125.66 ± 1.32	198.12 ± 9.30	< 0.001
**HDL-C (mg/dL)**	52.23 ± 0.18	46.03 ± 0.41	< 0.001
**LDL-C (mg/dL)**	115.71 ± 0.45	119.54 ± 1.17	0.002
**Non-HDL-C (mg/dL)**	140.40 ± 0.50	155.92 ± 1.60	< 0.001
**Numbers of dyslipidemia components**			< 0.001
0	5,153 (68.4)	459 (43.4)	
1	1,418 (17.9)	294 (27.2)	
2	941 (11.7)	243 (22.9)	
3 or more	149 (2.1)	65 (6.5)	
**Dyslipidemia medication**	958 (10.1)	113 (8.3)	0.102
**Hypertension**	2,321 (25.4)	484 (39.3)	< 0.001
**Diabetes**	951 (10.3)	158 (10.9)	< 0.001
**Current smoker**	1,177 (18.5)	301 (32.6)	< 0.001
**Alcohol consumption**	3,980 (56.8)	677 (69.0)	< 0.001
**Regular Exercise**	3,384 (47.4)	492 (51.3)	0.037

SBP, systolic blood pressure; DBP, diastolic blood pressure; BMI, body mass index; BUN, blood urea nitrogen; GFR, Glomerular filtration rate; hs-CRP, high sensitivity C-reactive protein; HDL-C, high density lipoprotein cholesterol; LDL-C, low density lipoprotein cholesterol.

Data are expressed as the weighted mean ± standard error or weighted percent (%).

^a^ p-values are calculated by Student’s t-test for continuous variables and Chi-square test for categorical variables.

### Association between the individual components of dyslipidemia and serum uric acid levels

**Fig 2:**
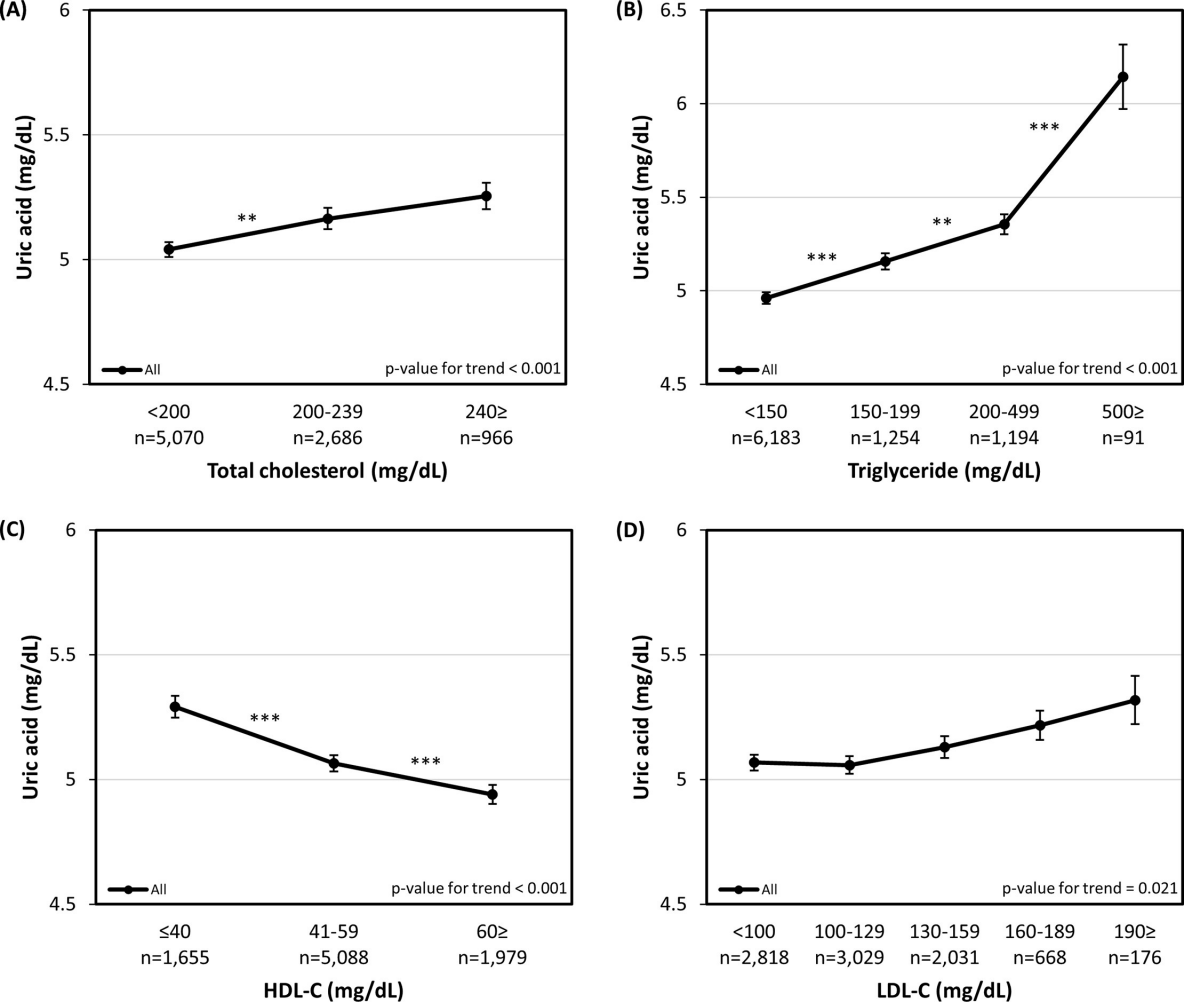
Adjusted serum uric acid levels according to the individual dyslipidemia components. (A) Total cholesterol (B) Triglyceride (C) HDL-C (D) LDL-C. Adjusted for age, sex, waist circumference, BMI, hemoglobin, BUN, GFR, HTN, DM, smoking, alcohol consumption, regular exercise and dyslipidemia medication (model 4). Error bars represent standard errors. Each number of asterisks corresponds to the following p-values. * p-value < 0.05, ** p-value < 0.01, *** p-value < 0.001.

Fig 2 represents the association between the individual components of dyslipidemia and serum uric acid levels in the whole participants group: (A) Total cholesterol (B) Triglyceride (C) HDL-C (D) LDL-C. After adjusting for age, sex, waist circumference, BMI, hemoglobin, BUN, GFR, hypertension, diabetes, smoking status, intake of alcohol, regular exercise and the administration of dyslipidemia medication (model 4), serum uric acid levels showed positive trend (or negative trend with HDL-C) within each components of dyslipidemia (P < 0.001) with the exception of LDL-C (P = 0.021). The analysis of non-HDL-C with hyperuricemia also showed consistent results of positive relationship, as presented in S1 Table (P < 0.001). When the analysis was further stratified according to sex, a more evident trend could be observed in the male group (S1 Fig).

**Fig 3:**
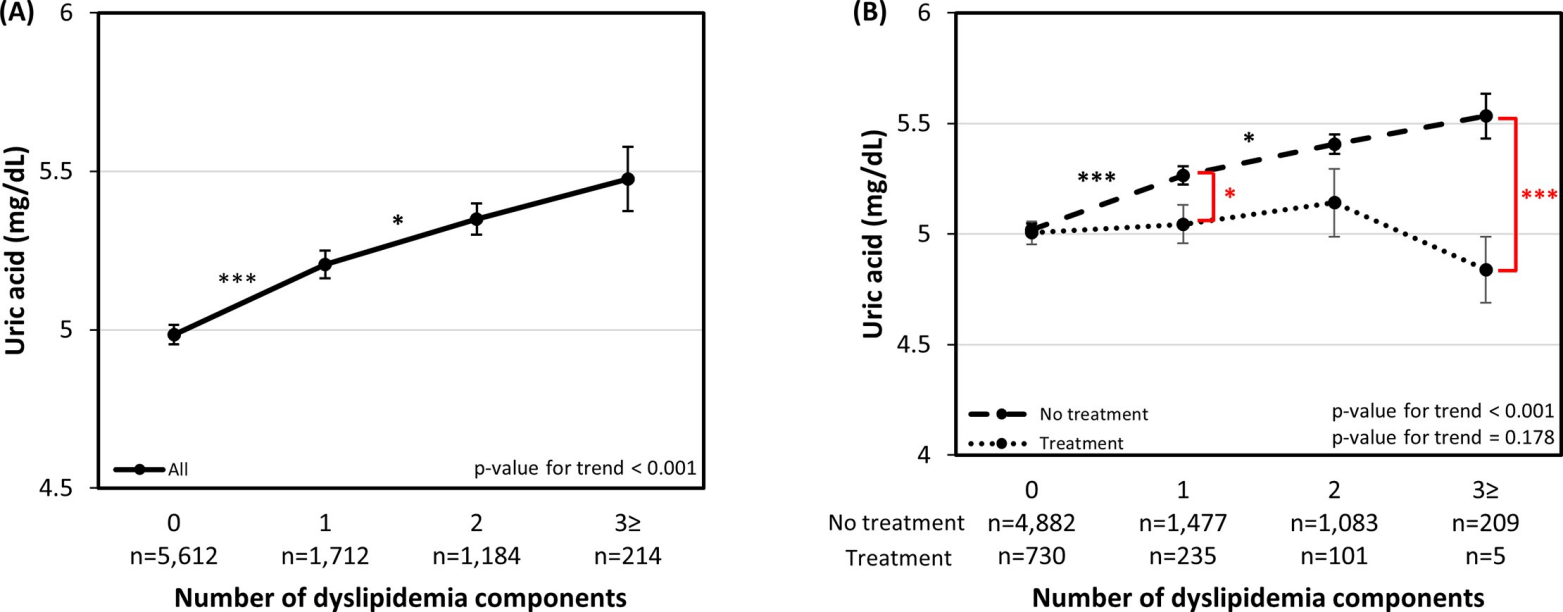
Adjusted serum uric acid levels according to the number of dyslipidemia components. (A) All participants, Adjusted for age, sex, waist circumference, BMI, hemoglobin, BUN, GFR, HTN, DM, smoking, alcohol consumption, regular exercise and dyslipidemia medication (model 4) (B) Dyslipidemia medication administered group and non-administered group, Adjusted for age, sex, waist circumference, BMI, hemoglobin, BUN, GFR, HTN, DM, smoking, alcohol consumption and regular exercise (model 3). Black asterisks are for comparison within-group and red asterisks are for comparison between groups. Error bars represent standard errors. Each number of asterisks corresponds to the following p-values. * p-value < 0.05, ** p-value < 0.01, *** p-value < 0.001.

The association between the increasing number of dyslipidemia components and serum uric acid levels is presented in Fig 3(A). For the all participants group, adjusted serum uric acid levels showed a tendency to increase concomitantly with the increasing number of dyslipidemia components, with each comparison statistically significant except with the one between components of two and three or more (P = 0.729).

Fig 3(B) shows the subgroup comparison of adjusted serum uric acid levels between the dyslipidemia medication administered group and the group without the administration of dyslipidemia medication. Since the groups were analyzed with regard to the administration of dyslipidemia medication, adjustments were performed with model 3. The results show that the uric acid level in the medication administrated group was significantly lower in the categories of one dyslipidemia component and the three or more dyslipidemia components (P < 0.05, P < 0.001, respectively).

### Relationship between the individual components of dyslipidemia and hyperuricemia

Table 2 presents the multivariable logistic regression analysis between the individual components of dyslipidemia and hyperuricemia in the all participants group. Odds ratios (OR) and 95% confidence interval (CI) of hyperuricemia to each of hypercholesterolemia, hypertriglyceridemia, HDL-C, and LDL-C levels were calculated. When adjusted for model 4, OR of all dyslipidemia components to hyperuricemia were shown to be statistically significant. For each 10 mg/dL increments of individual dyslipidemia components, the calculated OR for hyperuricemia retained statistical significance. Since LDL-C has a possibility for multicollinearity with other components due to the Friedewald formula, the further analysis takes into account only the total cholesterol levels, triglycerides and HDL-C levels. Individual components of hypercholesterolemia (OR 1.053; 95% CI 1.028–1.079, P < 0.001), hypertriglyceridemia (OR 1.017; 95% CI 1.009–1.026, P < 0.001) and HDL-C (OR 0.804; 95% CI 0.729–0.887, P < 0.001) retained significant correlations with hyperuricemia when further adjusted for the components combined (Table 3). Additionally, when non-HDL-C was replaced with total cholesterol, non-HDL-C (OR 1.050; 95% CI 1.023–1.077, P < 0.001) showed a significant correlation with hyperuricemia (S1 Table). The same analysis was made for the increasing numbers of dyslipidemia components, with the ORs of each cumulative numbers of one, two and three or more components statistically significant compared with the reference value of no dyslipidemia components (Table 3).

**Table 2:** Multivariable logistic regression analysis between the components of dyslipidemia and hyperuricemia.

		Crude			Model 1^a^			Model 2^b^			Model 3^c^			Model 4^d^		
	case (n)	OR	95% CI	p-value	OR	95% CI	p-value	OR	95% CI	p-value	OR	95% CI	p-value	OR	95% CI	p-value
**Total cholesterol**																
< 240 mg/dL	7,756	1			1			1			1			1		
≥ 240 mg/dL	966	1.686	1.367, 2.078	< 0.001	1.835	1.480, 2.276	< 0.001	1.619	1.300, 2.017	< 0.001	1.503	1.191, 1.897	0.001	1.455	1.152, 1.837	0.002
**Total cholesterol (10 mg/dL)**	8,722	1.069	1.046, 1.093	< 0.001	1.08	1.057, 1.104	< 0.001	1.064	1.041, 1.088	< 0.001	1.064	1.040, 1.088	< 0.001	1.059	1.035, 1.085	< 0.001
**Triglycerides**																
< 200 mg/dL	7,437	1			1			1			1			1		
≥ 200 mg/dL	1,285	3.438	2.915, 4.054	< 0.001	2.814	2.365, 3.349	< 0.001	2.264	1.894, 2.705	< 0.001	2.182	1.788, 2.664	< 0.001	2.154	1.763, 2.632	< 0.001
**Triglycerides (10 mg/dL)**	8,722	1.043	1.034, 1.052	< 0.001	1.035	1.026, 1.044	< 0.001	1.027	1.020, 1.035	< 0.001	1.028	1.020, 1.036	< 0.001	1.028	1.020, 1.036	< 0.001
**HDL-C**																
≤ 40 mg/dL	7,067	1			1			1			1			1		
> 40 mg/dL	1,655	0.39	0.335, 0.455	< 0.001	0.479	0.409, 0.563	< 0.001	0.578	0.490, 0.682	< 0.001	0.597	0.501, 0.710	< 0.001	0.605	0.508, 0.721	< 0.001
**HDL-C (10 mg/dL)**	8,722	0.631	0.584, 0.681	< 0.001	0.697	0.643, 0.755	< 0.001	0.776	0.714, 0.843	< 0.001	0.772	0.708, 0.842	< 0.001	0.775	0.711, 0.845	< 0.001
**LDL-C**																
< 160 mg/dL	7,878	1			1			1			1			1		
≥ 160 mg/dL	844	1.345	1.054, 1.717	0.017	1.57	1.214, 2.031	0.001	1.44	1.106, 1.876	0.007	1.384	1.045, 1.833	0.023	1.34	1.011, 1.777	0.042
**LDL-C (10 mg/dL)**	8,722	1.036	1.013, 1.060	0.002	1.05	1.025, 1.075	< 0.001	1.035	1.010, 1.060	0.005	1.037	1.012, 1.063	0.003	1.031	1.005, 1.057	0.02

OR, odds ratio; CI, confidence interval; BMI, body mass index; BUN, blood urea nitrogen; GFR, glomerular filtration rate; HTN, hypertension; DM, diabetes; HDL-C, high density lipoprotein cholesterol; LDL-C, low density lipoprotein cholesterol.

^a^ Model 1: Adjusted for sex and age.

^b^ Model 2: Adjusted for sex, age, and BMI.

^c^ Model 3: Adjusted for age, sex, waist circumference, BMI, hemoglobin, BUN, GFR, HTN, DM, smoking, alcohol consumption, and regular exercise.

^d^ Model 4: Adjusted for age, sex, waist circumference, BMI, hemoglobin, BUN, GFR, HTN, DM, smoking, alcohol consumption, regular exercise, and dyslipidemia medication.

**Table 3:** Multivariable logistic regression analysis between dyslipidemia and hyperuricemia.

		Crude			Model 1^a^			Model 2^b^			Model 3^c^			Model 4^d^		
	case (n)	OR	95% CI	p-value	OR	95% CI	p-value	OR	95% CI	p-value	OR	95% CI	p-value	OR	95% CI	p-value
**Combined lipid profile**	8,722															
Total cholesterol (10 mg/dL)		1.064	1.040, 1.087	< 0.001	1.07	1.046, 1.096	< 0.001	1.053	1.029, 1.078	< 0.001	1.053	1.028, 1.079	< 0.001	1.05	1.023, 1.077	< 0.001
Triglyceride (10 mg/dL)		1.022	1.013, 1.031	< 0.001	1.018	1.009, 1.027	< 0.001	1.017	1.009, 1.025	< 0.001	1.017	1.009, 1.026	< 0.001	1.017	1.009, 1.026	< 0.001
HDL-C (10 mg/dL)		0.675	0.619, 0.737	< 0.001	0.728	0.666, 0.796	< 0.001	0.806	0.736, 0.883	< 0.001	0.804	0.729, 0.887	< 0.001	0.808	0.732, 0.892	< 0.001
**Number of dyslipidemia components**	8,722															
0	5,612	1			1			1			1			1		
1	1,712	2.389	2.000, 2.853	< 0.001	2.068	1.724, 2.479	< 0.001	1.747	1.446, 2.111	< 0.001	1.702	1.389, 2.085	< 0.001	1.686	1.376, 2.065	< 0.001
2	1,184	3.092	2.552, 3.746	< 0.001	2.933	2.394, 3.594	< 0.001	2.365	1.926, 2.902	< 0.001	2.194	1.751, 2.748	< 0.001	2.146	1.712, 2.690	< 0.001
3 or more	214	4.99	3.539, 7.038	< 0.001	4.228	3.009, 5.940	< 0.001	3.096	2.197, 4.364	< 0.001	2.903	2.024, 4.165	< 0.001	2.813	1.956, 4.046	< 0.001

OR, odds ratio; CI, confidence interval; BMI, body mass index; BUN, blood urea nitrogen; GFR, glomerular filtration rate; HTN, hypertension; DM, diabetes; HDL-C, high density lipoprotein cholesterol.

^a^ Model 1: Adjusted for sex and age.

^b^ Model 2: Adjusted for sex, age, and BMI.

^c^ Model 3: Adjusted for age, sex, waist circumference, BMI, hemoglobin, BUN, GFR, HTN, DM, smoking, alcohol consumption, and regular exercise.

^d^ Model 4: Adjusted for age, sex, waist circumference, BMI, hemoglobin, BUN, GFR, HTN, DM, smoking, alcohol consumption, regular exercise, and dyslipidemia medication.

## Discussion

Our analysis of 8,722 participants from the KNHANES VII survey suggests that there is a significant relationship between the individual components of dyslipidemia and the serum uric acid levels. To the best of our knowledge, this is the first large-scale study that reports the strong association between the two factors in a nationally representative sample of Korean adults.

Several important implications can be drawn from the present study. First, total cholesterol, triglycerides, LDL-C and non-HDL-C levels were positively associated with serum uric acid levels. Second, there was an inverse relationship between HDL-C levels and serum uric acid. These relationships remained significant regardless of adjustment for known risk factors and confounding variables including age, sex, BMI and others. Third, when the analysis was further stratified according sex, a more evident trend of association could be observed in the male participants group. Fourth, the increasing numbers of dyslipidemia components have shown to be positively associated with serum uric acid levels. Fifth, a recognizable trend of lower serum uric acid levels was observed in the group administered with dyslipidemia medication, compared with serum uric acid levels in the non-administered group.

Hyperuricemia is now considered to be an important risk factor for hypertension, metabolic syndrome, chronic kidney disease and cardiovascular disease [20]. Serum uric acid, previously known to have a beneficial effect as an oxygen radical scavenger [21], is now recognized to carry a vital role as the modulator of glucose and lipid metabolism [22], leading to observations that the breach of serum uric acid homeostasis may be associated with cardiovascular risk and all-cause mortality [22–24]. Current studies demonstrate the possibility of serum uric acid level as a predictor of incident metabolic syndrome [25] and, when combined with LDL-C, of incident hypertension [26]. However, the independent role of serum uric acid as a novel risk factor and a target for treatment regardless of the clinical history of gout has been a subject of debate. Despite growing pieces of evidence, consensus on whether the treatment of asymptomatic hyperuricemia would be beneficial or not is yet to be established [22, 23, 27, 28].

In the present study, individual dyslipidemia components showed a statistically significant correlation with hyperuricemia even after adjusting for multiple laboratory & clinical confounders. Similar observations have been made in three previous cross-sectional studies [5, 8]. In the study by Peng et al., the serum LDL-C levels, triglycerides, total cholesterol, apolipoprotein-B levels, ratio of triglycerides to HDL-C and ratio of apolipoprotein-B to apolipoprotein-A1 were significantly associated with serum uric acid levels, whereas serum HDL-C levels were inversely associated [5]. The study by Ali et al. showed similar results to the aforementioned study with apolipoprotein-B and apolipoprotein-A1 not included in the data set [8]. Also, a study by Xu et al. has demonstrated that the parameter of non-HDL-C, along with triglycerides, is associated with hyperuricemia [29]. Our analysis has shown consistent results with the above findings.

The more distinct trend of male participants in the association between dyslipidemia and serum uric acid levels is worth noting. Studies to date have shown controversial results regarding whether gender differences mediate the association. A study by Stelmach et al. on Polish adults with hyperuricemia showed that the triglyceride values were higher in the upper tertile of serum uric acid levels in males but not in females [30]. A retrospective study by Lippi et al. demonstrated contradicting results, showing associations between triglycerides and serum uric acid levels in women but not in men [10]. In this regard, the results of our study demonstrate positive association between triglycerides and serum uric acid levels in both male and female groups.

Further investigations in our study include the positive association between the increasing number of dyslipidemia components and the serum uric acid levels. Considering the above discussion on the relationship of individual dyslipidemia components with serum uric acid, our findings demonstrate that individual components of dyslipidemia collectively and individually associate with serum uric acid levels.

Our comparison between dyslipidemia medication administered and non-administered subgroups showed that although not coherent in the category of two dyslipidemia components, a trend of lower serum uric acid levels in the medication administered group can be recognized. This result is in line with previous studies relating to statin and fenofibrate therapy to the decrease of serum uric acid levels [31–33].

The current study has some limitations and restricting elements. The cross-sectional study design does not warrant us to draw cause-effect relationships between the dyslipidemia components and serum uric acid levels. Also, the nature of epidemiological study using surveys with established sets of variables was of significant restraint. The lack of data on other lipid parameters (lipoprotein(a), apolipoprotein-A1, apolipoprotein-B) other than the parameters covered in this study should be considered as a relevant limitation. Important factors, such as which medications of lipid lowering agents were used, participant history of gout and whether urate lowering treatments were administered or not, weren’t included in the questionnaire, leading to insufficient data. However, considering the low prevalence of gout in the general Korean population (0.76%) [34], it is unlikely that the unavailable data regarding the clinical history of gout or the participants taking urate lowering agents significantly affected the results [12].

Whether serum uric acid is only a marker of preexisting disorder or a causal factor for dyslipidemia and cardiovascular disease remains controversial [5]. Regarding the relationship between serum uric acid and the lipoprotein metabolism, studies have shown that elevated serum uric acid is a significant predictor of smaller and denser LDL-C and HDL-C particles, which are known to be related with higher cardiovascular disease risk [35]. Although these previous studies and biological plausibility coherently support the results of this study, sufficiently powered prospective randomized clinical trials would be warranted for establishing causal relationships between the factors and for further applications into clinical practice.

## Conclusion

After adjusting for potential confounders, our study demonstrated that the dyslipidemia components of serum total cholesterol, triglycerides and LDL-C levels are positively associated with serum uric acid levels, whereas serum HDL-C levels are inversely related. Further complementary studies regarding other lipid parameters are needed to confirm the accurate association between dyslipidemia and serum uric acid levels.

## Supporting information

S1 Fig Adjusted serum uric acid levels in male & female group according to the individual dyslipidemia components. (A) Total cholesterol (B) Triglyceride (C) HDL-C (D) LDL-C. Adjusted for age, sex, waist circumference, BMI, hemoglobin, BUN, GFR, HTN, DM, smoking, alcohol consumption, regular exercise and dyslipidemia medication (model 4). Error bars represent standard errors. Each number of asterisks corresponds to the following p-values. * p-value < 0.05, ** p-value < 0.01, *** p-value < 0.001.
(DOCX)

S1 Table Multivariable logistic regression analysis between non-HDL-C and hyperuricemia. OR, odds ratio; CI, confidence interval; BMI, body mass index; BUN, blood urea nitrogen; GFR, glomerular filtration rate; HTN, hypertension; DM, diabetes; HDL-C, high density lipoprotein cholesterol. ^a^ Model 1: Adjusted for sex and age. ^b^ Model 2: Adjusted for sex, age, and BMI. ^c^ Model 3: Adjusted for age, sex, waist circumference, BMI, hemoglobin, BUN, GFR, HTN, DM, smoking, alcohol consumption, and regular exercise. ^d^ Model 4: Adjusted for age, sex, waist circumference, BMI, hemoglobin, BUN, GFR, HTN, DM, smoking, alcohol consumption, regular exercise, and dyslipidemia medication.
(DOCX)
