# Supplementary material for: Association between dyslipidemia and serum uric acid levels in Korean adults: Korea National Health and Nutrition Examination Survey 2016-2017
Source: PLoS One. 2020 Feb 14;15(2):e0228684. doi: 10.1371/journal.pone.0228684 (PMC7021293; doi:10.1371/journal.pone.0228684)

**S1 Fig. Adjusted serum uric acid levels in male & female group according to the individual dyslipidemia components.**

(A) Total cholesterol (B) Triglyceride (C) HDL-C (D) LDL-C. Adjusted for age, sex, waist circumference, BMI, hemoglobin, BUN, GFR, HTN, DM, smoking, alcohol consumption, regular exercise and dyslipidemia medication (model 4). Error bars represent standard errors. Each number of asterisks corresponds to the following p-values. * p-value < 0.05, ** p-value < 0.01, *** p-value < 0.001


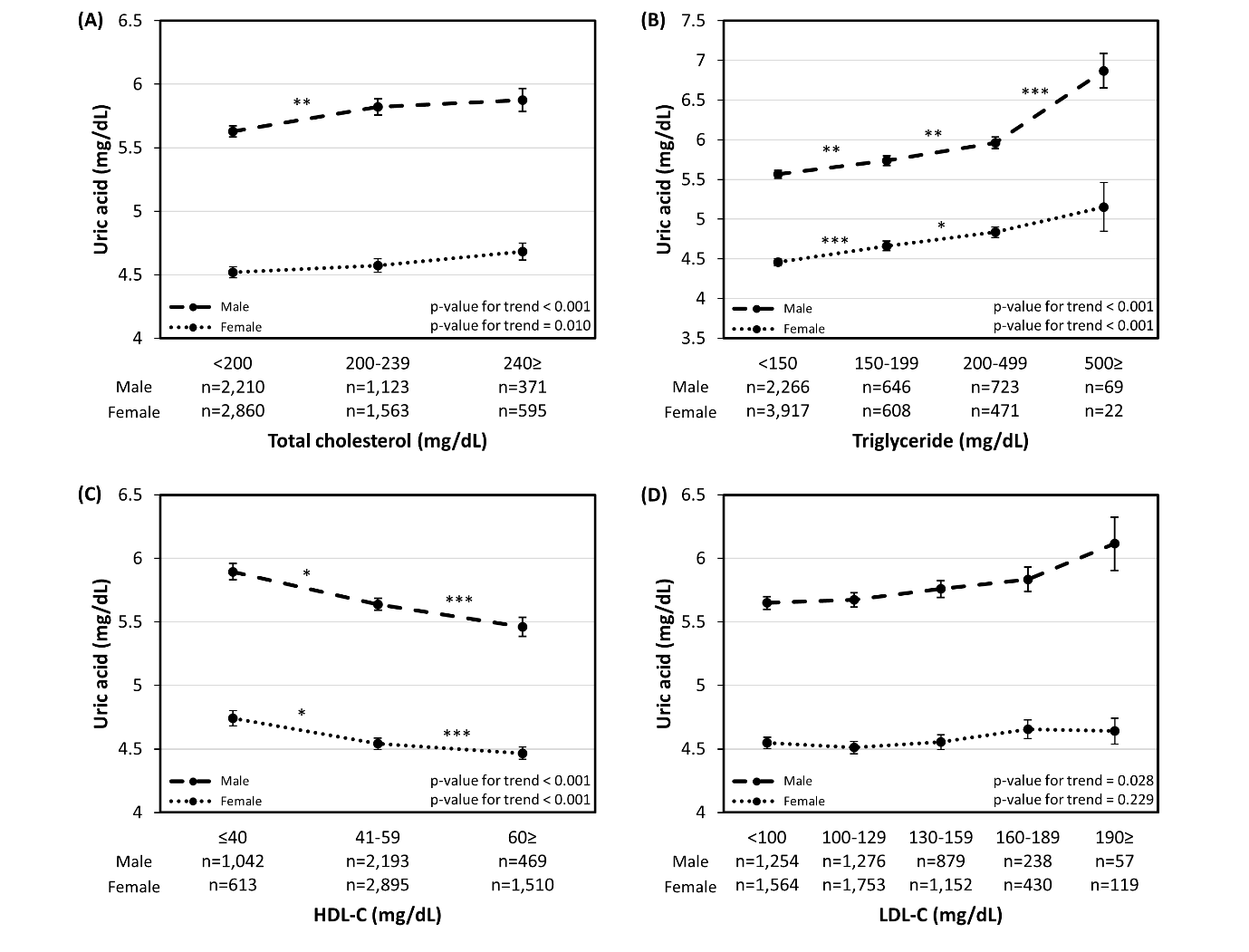

Supplement: S1 Fig — (A) Total cholesterol (B) Triglyceride (C) HDL-C (D) LDL-C. Adjusted for age, sex, waist circumference, BMI, hemoglobin, BUN, GFR, HTN, DM, smoking, alcohol consumption, regular exercise and dyslipidemia medication (model 4). Error bars represent standard errors. Each number of asterisks corresponds to the following p-values. * p-value < 0.05, ** p-value < 0.01, *** p-value < 0.001. (DOCX) [file pone.0228684.s001.docx]
