# Supplementary material for: Association between dyslipidemia and serum uric acid levels in Korean adults: Korea National Health and Nutrition Examination Survey 2016-2017
Source: PLoS One. 2020 Feb 14;15(2):e0228684. doi: 10.1371/journal.pone.0228684 (PMC7021293; doi:10.1371/journal.pone.0228684)
Supplement: S1 Table — OR, odds ratio; CI, confidence interval; BMI, body mass index; BUN, blood urea nitrogen; GFR, glomerular filtration rate; HTN, hypertension; DM, diabetes; HDL-C, high density lipoprotein cholesterol. a Model 1: Adjusted for sex and age. b Model 2: Adjusted for sex, age, and BMI. c Model 3: Adjusted for age, sex, waist circumference, BMI, hemoglobin, BUN, GFR, HTN, DM, smoking, alcohol consumption, and regular exercise. d Model 4: Adjusted for age, sex, waist circumference, BMI, hemoglobin, BUN, GFR, HTN, DM, smoking, alcohol consumption, regular exercise, and dyslipidemia medication. (DOCX) [file pone.0228684.s002.docx]

**S1 Table. Multivariable logistic regression analysis between non-HDL-C and hyperuricemia.**

|  |  | **Crude** | | | **Model 1^a^** | | | **Model 2^b^** | | | **Model 3^c^** | | | **Model 4^d^** | | |
| --- | --- | --- | --- | --- | --- | --- | --- | --- | --- | --- | --- | --- | --- | --- | --- | --- |
|  | **case (n)** | OR | 95% CI | p-value | OR | 95% CI | p-value | OR | 95% CI | p-value | OR | 95% CI | p-value | OR | 95% CI | p-value |
| **Non-HDL-C** |  |  |  |  |  |  |  |  |  |  |  |  |  |  |  |  |
| < 160 mg/dL | 6,086 | 1 |  |  | 1 |  |  | 1 |  |  | 1 |  |  | 1 |  |  |
| ≥ 160 mg/dL | 2,636 | 2.149 | 1.844, 2.504 | < 0.001 | 2.076 | 1.765, 2.441 | < 0.001 | 1.767 | 1.501, 2.081 | < 0.001 | 1.710 | 1.433, 2.039 | < 0.001 | 1.665 | 1.385, 2.001 | < 0.001 |
| **Non-HDL-C**  **(10 mg/dL)** | 8,722 | 1.117 | 1.094, 1.139 | < 0.001 | 1.113 | 1.090, 1.137 | < 0.001 | 1.087 | 1.064, 1.111 | < 0.001 | 1.088 | 1.063, 1.112 | < 0.001 | 1.085 | 1.059, 1.111 | < 0.001 |
| **Combined**  **lipid profile** | 8,722 |  |  |  |  |  |  |  |  |  |  |  |  |  |  |  |
| Non-HDL-C  (10 mg/dL) |  | 1.064 | 1.040, 1.087 | < 0.001 | 1.070 | 1.046, 1.096 | < 0.001 | 1.053 | 1.029, 1.078 | < 0.001 | 1.053 | 1.028, 1.079 | < 0.001 | 1.050 | 1.023, 1.077 | < 0.001 |
| Triglyceride  (10 mg/dL) |  | 1.022 | 1.013, 1.031 | < 0.001 | 1.018 | 1.009, 1.027 | < 0.001 | 1.017 | 1.009, 1.025 | < 0.001 | 1.017 | 1.009, 1.026 | < 0.001 | 1.017 | 1.009, 1.026 | < 0.001 |
| HDL-C  (10 mg/dL) |  | 0.718 | 0.661, 0.781 | < 0.001 | 0.779 | 0.715, 0.849 | < 0.001 | 0.849 | 0.778, 0.926 | < 0.001 | 0.847 | 0.773, 0.929 | < 0.001 | 0.848 | 0.774, 0.930 | < 0.001 |

OR, odds ratio; CI, confidence interval; BMI, body mass index; BUN, blood urea nitrogen; GFR, glomerular filtration rate; HTN, hypertension; DM, diabetes; HDL-C, high density lipoprotein cholesterol.

^a^ Model 1: Adjusted for sex and age.

^b^ Model 2: Adjusted for sex, age, and BMI.

^c^ Model 3: Adjusted for age, sex, waist circumference, BMI, hemoglobin, BUN, GFR, HTN, DM, smoking, alcohol consumption, and regular exercise.

^d^ Model 4: Adjusted for age, sex, waist circumference, BMI, hemoglobin, BUN, GFR, HTN, DM, smoking, alcohol consumption, regular exercise, and dyslipidemia medication.
